# Lactate-mediated glia-neuronal signalling in the mammalian brain

**DOI:** 10.1038/ncomms4284

**Published:** 2014-02-11

**Authors:** F. Tang, S. Lane, A. Korsak, J. F. R. Paton, A. V. Gourine, S. Kasparov, A. G. Teschemacher

**Affiliations:** 1 School of Physiology and Pharmacology, University of Bristol, Bristol BS8 1TD, UK; 2 Department of Neuroscience, Physiology & Pharmacology, University College London, London WC1E 6BT, UK; 3 These authors contributed equally to this work

## Abstract

Astrocytes produce and release L-lactate as a potential source of energy for neurons. Here we present evidence that L-lactate, independently of its caloric value, serves as an astrocytic signalling molecule in the locus coeruleus (LC). The LC is the principal source of norepinephrine to the frontal brain and thus one of the most influential modulatory centers of the brain. Optogenetically activated astrocytes release L-lactate, which excites LC neurons and triggers release of norepinephrine. Exogenous L-lactate within the physiologically relevant concentration range mimics these effects. L-lactate effects are concentration-dependent, stereo-selective, independent of L-lactate uptake into neurons and involve a cAMP-mediated step. *In vivo* injections of L-lactate in the LC evokes arousal similar to the excitatory transmitter, L-glutamate. Our results imply the existence of an unknown receptor for this ‘glio-transmitter’.

## Abstract (web summary)

The astrocytic release of the metabolite L-lactate is implicated in modulating neuronal activity in the brain. Here, the authors show that L-lactate released from astrocytes excites noradrenergic neurons in the locus coeruleus and triggers the release of noradrenaline, increasing network excitability.

## Graphical abstract

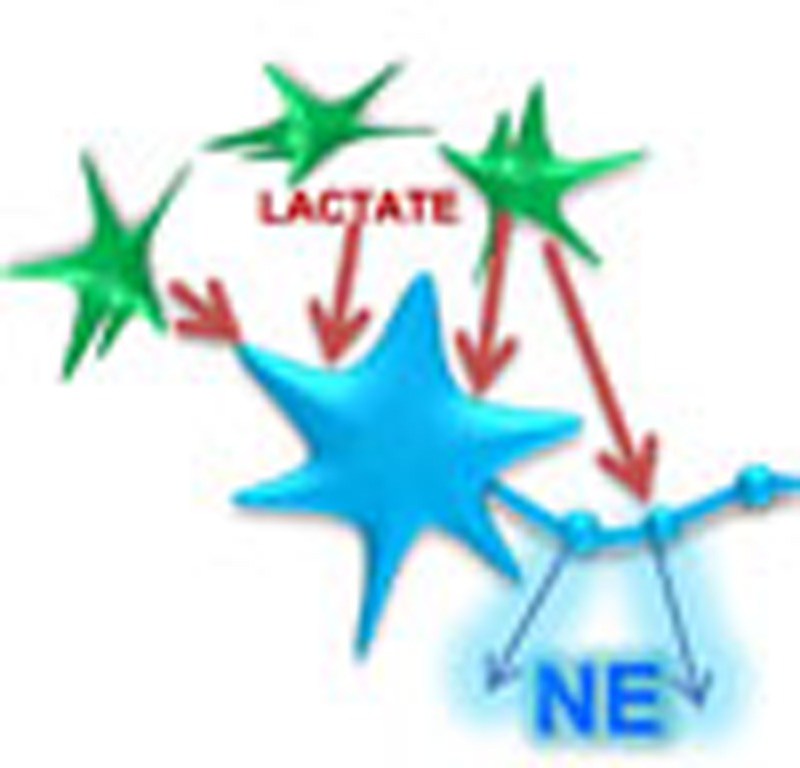

Astrocytes have been implicated in modulation of diverse neuronal circuits^1^,^2^,^3^,^4^. They influence activity of adjacent neurons in a number of ways, including fine tuning the extracellular K^+^ concentration^1^, but by far the best-studied modulatory mechanism involves release of ‘glio-transmitters’—signalling molecules such as ATP^5^,^6^, glutamate^7^ or D-serine^8^. Yet another mechanism through which astrocytes may affect neuronal activity is commonly referred to as the ‘astrocyte-to-neuron lactate shuttle’^9^,^10^,^11^. In contrast to neurons, astrocytes are ideally poised to serve as a source of L-lactate, as they are capable of storing glucose as glycogen, which they can rapidly mobilize and direct to the glycolytic pathway^12^,^13^. This allows for highly dynamic local concentrations of L-lactate. According to the ‘lactate shuttle’ hypothesis, L-lactate released by astrocytes is taken up by neurons via monocarboxylate transporters (MCT) and used as a metabolic substrate, possibly in preference to glucose. Interfering with the ‘shuttle’ using various pharmacological approaches, for example 1,4-dideoxy-1,4-imino-D-arabinitol (DAB), affects long-term potentiation and memory^14^. The L-lactate stereoisomer, D-lactate (D-lactate) impairs memory in chick, and in the rat, presumably via antagonism with L-lactate^14^,^15^. At the same time, there is a considerable body of evidence against the idea that neurons prefer L-lactate to glucose as their energy source^12^,^16^. In summary, there is a consensus that astrocytes can release L-lactate upon various stimuli and that L-lactate may affect neuronal function, but the mechanism of L-lactate action remains unclear.

The intimate links between astrocytes and neurons that release norepinephrine (NE) were recognized long ago^17^,^18^ but the possibility that astrocytes may control the activity of these neurons and, by doing so, affect brain functions has not been explored. The largest group of NE neurons is the A6 group or locus coeruleus (LC), which supplies NE to the majority of forebrain structures. Interestingly, the input from LC does not only affect neurons but also cortical astrocytes^19^. The LC has been implicated in control of sleep-wakefulness state, vigilance, appetite, respiration, emotions and autonomic outflows. It may, therefore, be postulated that if astrocytes are able to modulate the activity of LC noradrenergic (NEergic) neurons and affect the release of NE, such a mechanism should have major physiological implications.

Previously, we reported that the astrocytes located on the ventral surface of the medulla oblongata play a role in central chemosensitivity and could be a component of the central homeostate, which maintains constant levels of *P*CO_2_/pH^4^. In that study, we established an optogenetic approach to investigate astrocyte-to-neuron communication using gene expression targeted to either astrocytes or specific neuronal phenotypes. Here, we apply similar strategies to study signalling between astrocytes and LC neurons. We stimulate astrocytes expressing optogenetic actuators with light and measure electrophysiological responses in LC neurons using patch clamp and also directly determine release of NE with fast-scan cyclic voltammetry (FCV). FCV assesses the concentration of NE by measuring its oxidation at a carbon fiber electrode charged to a specific potential. These experiments reveal that L-lactate, released by activated astrocytes or applied by bath, has a powerful excitatory effect on LC neurons, triggering release of NE. Moreover, application of L-lactate to the LC *in vivo* elicits responses that are qualitatively similar to those evoked by the prototypical excitatory neurotransmitter L-glutamate (GLU). The characteristics of the L-lactate effect on LC neurons may not be explained by its use as a metabolic substrate, but instead support the novel idea that L-lactate acts as a true ‘glio-transmitter’.

## Results

### L-lactate is involved in astrocyte to neuron signalling in LC

**Figure 1:**
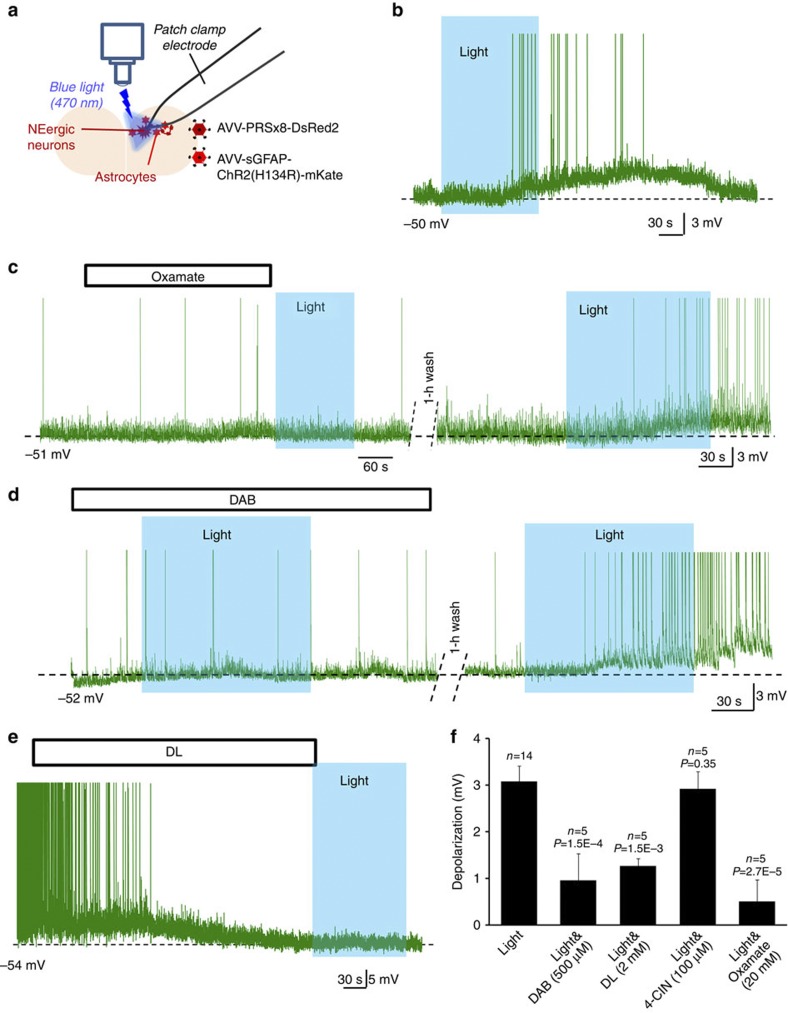
L-lactate mediates excitation of LC neurons following activation of local astrocytes. (**a**) Schematic to illustrate the approach. In rat organotypic-cultured slices containing LC, NEergic neurons were labelled by AVV-driven expression of DsRed2 protein under control of the PRSx8 promoter, which restricts expression to these cells. Two days later, slices were additionally transduced with an AVV to express ChR2(H134R)-mKate in astrocytes. This allowed visualization and patch recording of LC neurons using green or yellow laser lines of a confocal microscope without strong activation of ChR2(H134R). For stimulation, a 470 nm beam from a light emitting diode was deflected into the light path with an additional mirror. A similar approach was also used for [Ca^2+^]_i_ imaging with Rhod-2, as in our previous work^4^. (**b**) Application of flashing blue light reliably led to depolarization and increased firing of LC neurons, irrespective of whether cells were silent in control or had on-going activity. The latency of this response was markedly longer than could be expected from the [Ca^2+^]_i_ dynamics in ChR2(H134R)-expressing astrocytes^4^. Note that in these and most other example traces action potentials are truncated. (**c**) Inhibition of L-lactate synthesis by oxamate (20 mM) suppressed activation of an LC neuron evoked by optogenetic excitation of astrocytes. A depolarization could be evoked in the same cell following wash out of oxamate. (**d**) Inhibition of glycogen mobilization by DAB (500 μM) suppressed activation of an LC neuron evoked by optogenetic excitation of astrocytes. A depolarization could be evoked in the same cell following wash out of DAB. (**e**) D-lactate hyperpolarized LC neurons and inhibited light-induced depolarizations. (**f**) Pooled data demonstrating the involvement of L-lactate in astrocyte to neuron signalling in the LC. Error bars show s.e.m. All comparisons are against effect of optical stimulation without drugs (Student’s unpaired *t*-test).

**Figure 2:**
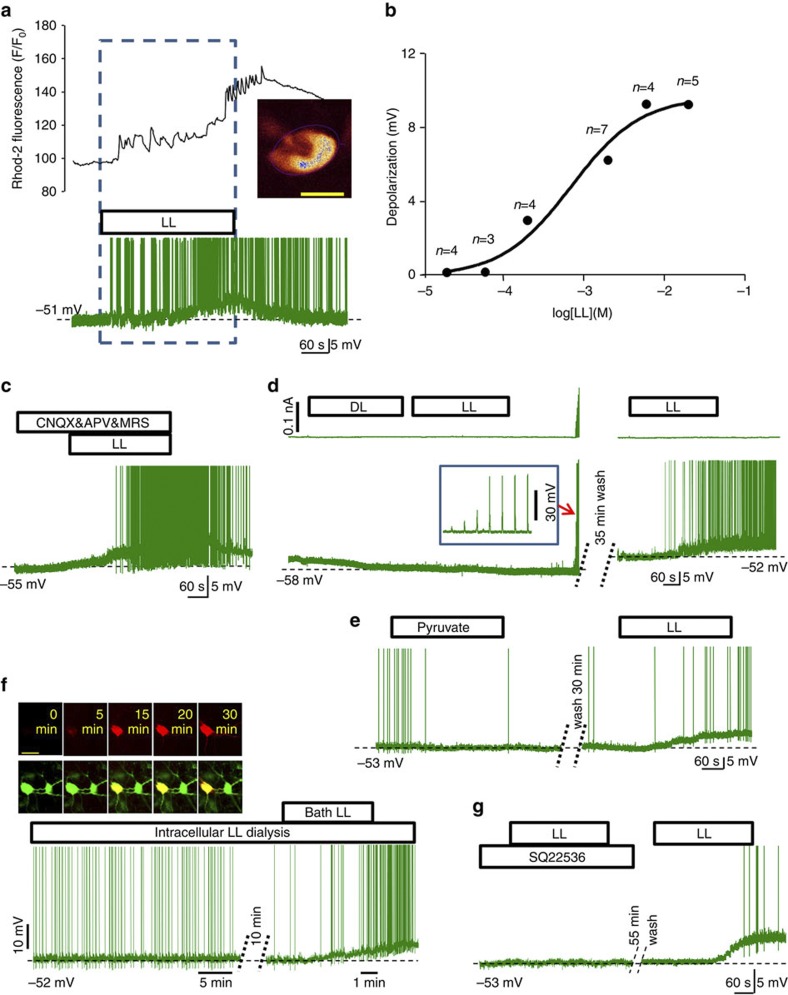
Exogenous L-lactate excites LC neurons. (**a**) Example of changes in [Ca^2+^]_i_ (upper panel) and membrane potential (lower panel) evoked by bath application of L-lactate in a patched LC neuron (inset, note that this is a single confocal plane, image is pseudo-coloured, scale bar, 15 μm). (**b**) Concentration-response curve for L-lactate-evoked depolarizations predicts an EC_50_ of ~680 μM L-lactate (as calculated by GraphPad Prizm). (**c**) A mix of glutamate and P2 receptor antagonists (CNQX, APV, MRS2179) does not affect the ability of L-lactate to excite LC neurons, arguing against an indirect network effect of L-lactate. (**d**) D-lactate (2 mM) completely blocks the excitatory effect of L-lactate (2 mM), while the cell is responsive and generates robust action potentials upon current injections (upper trace and inset). After 35 min of washing out D-lactate, L-lactate (2 mM) is able to evoke the characteristic excitation, demonstrating the reversibility of this antagonism. (**e**) Pyruvate that is transported into cells via the same mechanisms as L-lactate is completely without effect. L-lactate applied after a recovery period causes characteristic excitation. (**f**) Intracellular dialysis of L-lactate has no effect on the on-going activity of an LC neuron but bath-applied L-lactate is still excitatory. The upper image illustrates gradual diffusion of a solution containing L-lactate (2 mM), contrasted with a red dye (SNARF-5), into the patched cell. Scale bar, 35 μm. The lower trace (note the time scale) demonstrates the absence of any time-dependent changes in cellular activity. Application of extracellular L-lactate by superfusion elicits characteristic depolarization and action potentials. (**g**) SQ22536 (100 μM) completely blocks the excitatory effect of L-lactate (2 mM). Following wash out of SQ22536, L-lactate (2 mM) is able to evoke the characteristic excitation, demonstrating the reversibility of this inhibition.

Initially, we used the same *in vitro* experimental paradigm in cultured slices as in our previous work^4^ to study interactions between local astrocytes and LC neurons. A few additional tests were performed in primary dissociated cultured astrocytes as specified below. For electrophysiological recordings, astrocytes were transduced with an adenoviral vector (AVV) to express a Channelrhodopsin-2 mutant, ChR2(H134R), fused to red monomeric mKate for visualization (Fig. 1a) or with optoβ_2_ adrenergic receptors (optoβ_2_AR), which, upon activation, initiate intracellular signalling cascades similar to those evoked by endogenous astrocytic β_2_-adrenergic receptors^18^,^20^. Qualitatively both optogenetic constructs yielded similar results (Supplementary Fig. 1a–c). ChR2(H134R) was much easier to use in patch clamp experiments probably because of its lower sensitivity to light and hence was used in experiments presented in Fig. 1. NEergic neurons were targeted to express DsRed2 with AVV.sPRSx8.DsRed2 (Fig. 1a). They were visualized using yellow light through confocal optics and recorded in whole-cell mode. Some LC neurons under these conditions were silent, while others showed slow ongoing activity of 1–2 Hz (Figs 1 and 2). Their resting membrane potential was in the same range as previously found in acute slices^21^,^22^. Astrocytes then were activated with episodes of flashing 470 nm light. In all cells recorded, optogenetic stimulation of astrocytes evoked depolarization and increased action potential firing of NEergic neurons (Fig. 1b–f). We noted a significant latency of this response (typically over 60 s) and hypothesized that it may reflect recruitment of an enzymatic process and generation of an intermediate such as L-lactate. Indeed, inhibition of L-lactate dehydrogenase by oxamate^16^ to prevent conversion of pyruvate into L-lactate reversibly prevented light-induced depolarizations (Fig. 1c,f). Blockade of glycogen metabolism with DAB (500 μM)^23^ had a similar effect (Fig 1d,f). We reasoned that L-lactate produced in response to optogenetic stimulation of glial cells should lead to their intracellular acidification. This was confirmed in pure dissociated astrocytic cultures loaded with the pH indicator SNARF-5AM (Supplementary Fig. 2a). Moreover, such acidification could be prevented by pre-incubation with DAB (500 μM), consistent with involvement of an acidic product of glycogen mobilization. Note that similar results were obtained by activating astrocytes using optoβ_2_AR (Supplementary Fig. 2b,c)

D-lactate has been used previously to antagonize L-lactate actions in a number of behavioural studies^14^,^15^. Bath-applied D-lactate (2 mM) hyperpolarized LC neurons by 5.52±1.65 mV (±s.e.m.; *n*=6; *P*=0.0013; Student’s paired *t*-test; Fig. 1e) and almost completely abrogated depolarizations evoked by activation of astrocytes (Fig. 1e,f). Surprisingly, the MCT inhibitor 4-CIN (100 μM, concentration previously reported to block L-lactate import into central neurons^14^,^24^) was unable to prevent neuronal responses to astrocytic activation (Fig. 1f). This prompted us to evaluate effects of exogenously applied L-lactate on the activity of LC neurons.

### Exogenous L-lactate is excitatory to LC neurons

**Figure 3:**
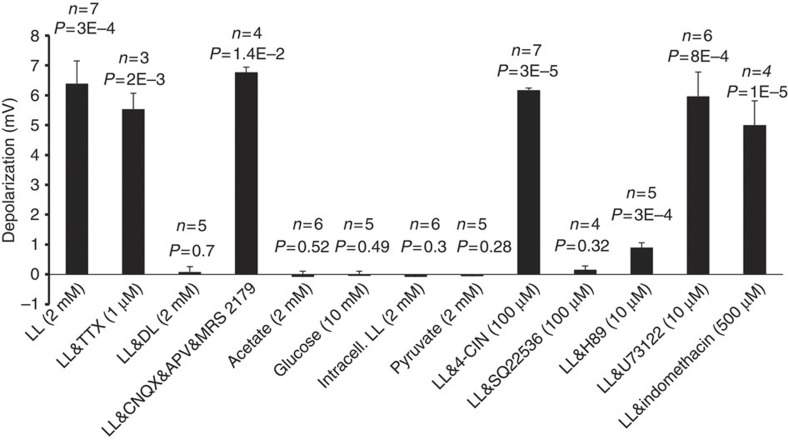
Mechanism of excitation of LC neurons by exogenous L-lactate. For all tests shown here 2 mM L-lactate was used. All comparisons are to their own baselines (Student’s paired *t*-test). Error bars depict s.e.m. Block of action potentials with TTX, of synaptic excitation with CNQX&APV&MRS2179, of monocarboxylase transporters with 4-CIN, of PLC with U73122 and of cyclo-oxygenase with indomethacin did not prevent depolarizations evoked by bath-applied L-lactate. However, D-lactate, SQ22536 and H89 suppressed them. Although a slight depolarization could still be detected in the presence of H89, the effect of L-lactate was drastically reduced (compare to the leftmost bar, *P*<0.001, Student’s unpaired *t*-test). Acetate (2 mM), increased extracellular glucose (from 2.5 to 10 mM), intracellular L-lactate (2 mM) and pyruvate (2 mM) had no effect on the membrane potential of LC neurons.

Pharmacological analysis of the depolarizing effect of L-lactate is shown in Fig. 2 and summarized in Fig. 3. Bath application of L-lactate (0.2–20 mM; all solutions at pH 7.4) to organotypic slices resulted in reversible depolarization and increased rate of action potential firing in LC neurons, accompanied by marked increases in [Ca^2+^]_i_ (Fig. 2a,b). The depolarizing effect of L-lactate became significant at 0.2 mM and saturated at concentrations over 6 mM (Fig. 2b) with an apparent EC_50_ of 680 μM. Of note, [Ca^2+^]_i_ elevations and depolarizations in LC neurons were preserved in the presence of tetrodotoxin (TTX; 1 μM; Fig. 3; Supplementary Fig. 3). Depolarizations were also unaffected in the presence of a mixture of ionotropic GLU receptor antagonists and a P2 receptor antagonist (CNQX 10 μM, APV 50 μM, MRS2179 10 μM; Figs 2c and 3), indicating that L-lactate excites LC neurons directly. The action of L-lactate was stereo-selective. As in the previous experiments, 2 mM D-lactate hyperpolarized LC neurons and completely occluded the depolarization evoked by 2 mM L-lactate (Figs 2d and 3). D-lactate (200 μM) only slightly hyperpolarized LC neurons (−0.74±0.14 mV, *n*=3,±s.e.m.; *P*=0.0328, Student’s paired *t*-test) but significantly reduced the effect of 2 mM L-lactate (2.63±0.13 mV, *n*=3, ±s.e.m.; *P*=0.023, Student’s unpaired *t*-test; Supplementary Fig. 4a).

### Effect of L-lactate on LC neurons is not metabolic

All recorded neurons were patched with pipettes containing 2 mM ATP, 1 mM GTP and 5 mM glucose. The standard perfusion media contained 5.5 mM glucose and varying it between 2 mM and 10 mM had no effect on membrane potential or firing rate of LC cells (Fig. 3; Supplementary Fig. 4b). Under such conditions, it was extremely unlikely that the excitatory effect of L-lactate could be explained by its caloric value.

Several specifically designed experiments consistently demonstrated that the excitatory effect of L-lactate was not due to its uptake by LC neurons and use as an energy substrate. First, pyruvate (2 mM), which can also be transported into the cells by MCT^25^ and enter the Krebs cycle, had no effect (Figs 2e and 3). Second, when 2 mM L-lactate was added to the patch pipette solution, membrane potential or spiking activity of LC neurons did not change over the period of up to 30 min after establishing whole-cell configuration (Figs 2f and 3). This time was enough to dialyze these neurons as evident from the diffusion of a red fluorescent dye (Fig. 2f inset). However, when the same neurons were later challenged by bath-applied L-lactate, it had a powerful excitatory effect (Fig. 2f). Third, because our recordings are usually performed in HEPES-buffered solution (HBS), we checked whether a switch to a 95% O_2_/5% CO_2_ saturated bicarbonate-based artificial cerebrospinal fluid (composition as per^26^) affects responses to L-lactate. However, the L-lactate effect was similar to the one registered in HBS (bicarbonate-based media: depolarization +6.46±0.75 mV, *n*=4; in HBS: 6.39±0.75 mV, *n*=7; ±s.e.m.; *P*=0.96, Student’s unpaired *t*-test). Fourth, the MCT blocker 4-CIN (100 μM) had no effect on the ability of L-lactate to activate LC neurons (Fig. 3), consistent with the idea that L-lactate does not need to enter LC neurons to excite them. Acetate (pH 7.4) which is thought to have high membrane permeability and be metabolizable did not mimic the excitatory effect of L-lactate (Fig. 3; Supplementary Fig. 4c).

Together these data suggest that L-lactate is not used by LC neurons as a source of energy but acts on a substrate located on the extracellular side of their plasma membrane to excite them.

### Depolarizations are mediated by protein kinase A signalling

L-lactate excites LC neurons without entering them, its effects should require an intracellular second messenger. We therefore tested the involvement of cAMP using an adenylate cyclase (AC) inhibitor, SQ22536 (100 μM) and found that it abolished the depolarizing effect of 2 mM L-lactate (Figs 2g and 3; Supplementary Fig. 4d). Many of the effects of cAMP are mediated by protein kinase A (PKA). The PKA inhibitor H89 (10 μM) suppressed depolarizations, but the phospholipase C (PLC) inhibitor U73122 (10 μM; Fig. 3) had no effect. It has been reported that L-lactate may interfere with uptake of prostaglandins^27^, leading to their accumulation, which could result in the effects described above. However, pre-incubation of slices with the cyclo-oxygenase inhibitor indomethacin (500 μM) for 30 min (ref. 28) did not significantly alter the effect of L-lactate (Fig. 3).

### L-lactate triggers NE release from cultured and acute slices

**Figure 4:**
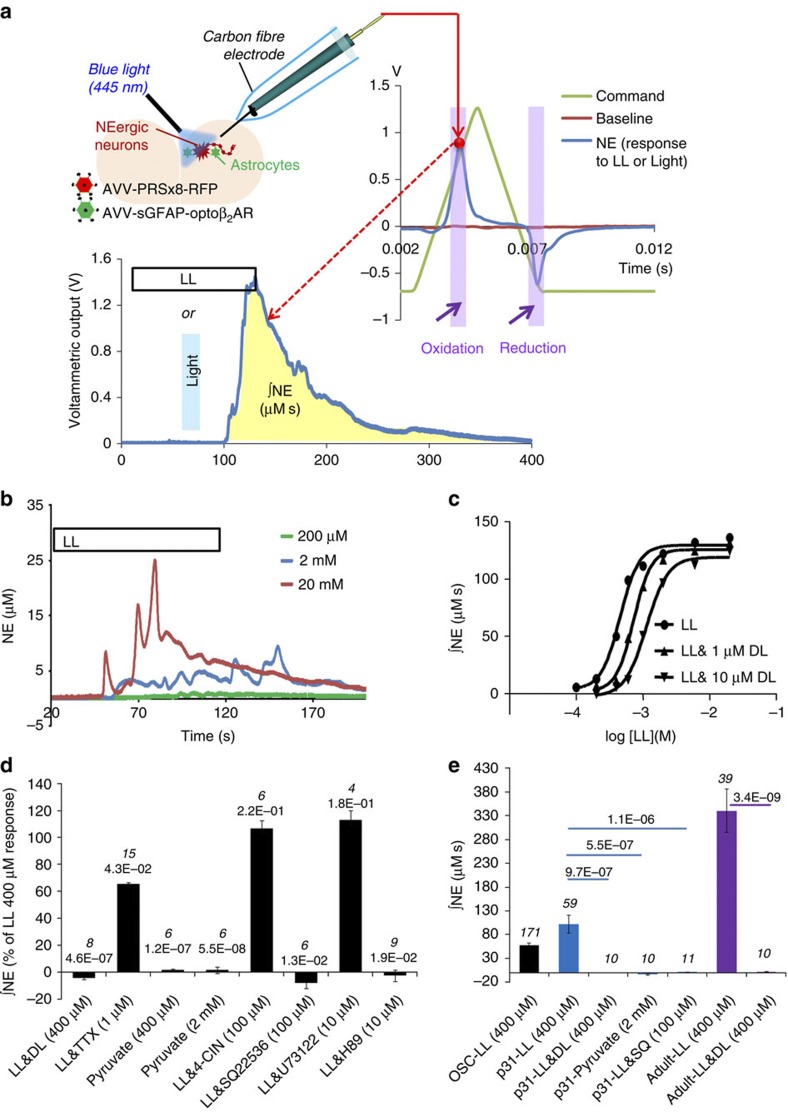
Fast scan cyclic voltammetry demonstrates that L-lactate is a powerful stimulant of NE release. (**a**) Schematic to illustrate the protocol. Left upper—organotypic brain slices containing the LC were transduced with AVV. AVV.sPRSx8.DsRed was used to visualize NEergic neurons with red fluorescence, AVV.sGFAP.optoβ_2_AR for optogenetic activation of G_s_PCR signalling in astrocytes. AVV.sPRSx8.ChIEF-tdTomato (see Supplementary Fig. 5) was used in some initial experiments to ascertain that optogenetic activation of NEergic neurons results in measurable NE release. Right—triangular voltage commands were applied via a carbon fibre electrode placed in the LC area. Currents at the typical oxidation potential for NE (~+0.65 to +0.85 V) were evaluated on sweep by sweep basis and used to calculate voltammetric output—left lower trace. The effect of stimulation was derived from the area under the curve post stimulus, which is thought to be proportional to the quantity of oxidized NE. After experiments, electrodes were calibrated against 0.5 μM NE in the recording chamber. (**b**) Examples of NE release evoked by bath application of L-lactate (0.2, 2, 20 mM) show that the effect is concentration dependent. (**c**) The concentration-response relationship for L-lactate (*n*=5 for each point) predicts an EC_50_ of 456 μM. The curve is shifted to the right with rising concentrations of D-lactate (1, 10 μM; *n*=5 for each data point, further detail in the text). (**d**) NE release evoked by L-lactate (0.4 mM) was blocked by D-lactate (0.4 mM; L-lactate: 35.3±4.3 μM s, *n*=14; L-lactate and D-lactate: −1.5±0.5 μM s, *n*=8), SQ22536 (100 μM; L-lactate: 35.0±11.0 μM s, *n*=5; L-lactate&SQ22536: −2.7±1.5 μM s, *n*=3) and by H89 (10 μM for >1 h; L-lactate: 107.6±36.5 μM s, *n*=5; L-lactate&H89: −2.8±4.7 μM s, *n*=9). The L-lactate effect was attenuated by TTX (1 μM; L-lactate: 120.4±22.8 μM s, *n*=20; L-lactate&TTX: 79.0±1.2 μM s, *n*=15) but not affected by 4-CIN (100 μM; L-lactate: 34.2±2.1 μM s, *n*=12; L-lactate&4-CIN: 36.5±2.0 μM s, *n*=6) or U73122 (10 μM; L-lactate: 49.2±5.6 μM s, *n*=5; L-lactate&U73122: 55.6±3.5 μM s, *n*=4). Pyruvate did not trigger NE release (pyruvate 400 μM: 1.7±0.6 μM s, *n*=6; pyruvate 2 mM: 1.6±2.5 μM s, *n*=6). Error bars depict s.e.m.; *P*-values are given above each data column; Student’s unpaired one-tailed *t*-test. (**e**) The effect of L-lactate is not limited to organotypic slice cultures (OSC). L-lactate was a strong stimulant of NE release also in acute LC slices of p31 and adult (~200 g) rats. In acute slices, D-lactate, pyruvate and SQ22536 had equivalent actions as in slice cultures. Error bars depict s.e.m., while *n* values are given above each data column. *P*-values are given above brackets comparing data columns; Student’s unpaired one-tailed *t*-test.

On the basis of the data described above L-lactate may be expected to stimulate release of NE. This was tested using FCV in organotypic cultured slices cut at the level of the LC (Fig. 4a). To initially confirm that LC neurons release NE upon activation, we expressed in LC neurons an optogenetic actuator ChIEF using AVV.sPRSx8.ChIEF-tdTomato, which also allowed visualization of transgene expressing neurons^29^,^30^. Carbon fibre electrodes placed in transduced slices detected clear time-locked increases in NE oxidation currents following trains of blue light pulses (1 ms pulses, 32 Hz for 10 s, power 2–6 mW mm^−2^). These currents were potentiated by a NE uptake inhibitor desipramine (DES; 500 nM), confirming the catecholamine nature of the signal (Supplementary Figs 5a and 6). The latency of these responses was ~30 s, indicating that the electrodes registered bulk accumulation of extracellular NE, which had to diffuse and possibly cross cellular membranes to reach the electrode.

Bath application of L-lactate (0.2–20 mM) markedly increased NE oxidation currents (Fig. 4b,c). The effect developed within 10–60 s from L-lactate arriving to the slice and was reversible and reproducible. The threshold L-lactate concentration was ~0.2 mM L-lactate, similar to what was found in patch clamp experiments (Fig. 2b). The concentration-response curve predicted an EC_50_ value of 456 μM (Fig. 4c). The effect of L-lactate was stereo-selective. D-lactate (400 μM) did not trigger NE release but completely blocked the stimulatory action of L-lactate (Fig. 4d). D-lactate (1 μM and 10 μM) shifted the L-lactate concentration-response curve to the right (Fig. 4c) resulting in EC_50_ of 728 μM (1 μM D-lactate) and 1.133 mM (10 μM D-lactate). TTX (1 μM) only mildly attenuated L-lactate-evoked release of NE (Fig. 4d). Therefore, in addition to somatic depolarization, L-lactate probably has a direct effect on the release machinery, which is partially action potential-independent. Note that, even in the presence of TTX, L-lactate still induced [Ca^2+^]_i_ elevations and depolarizations in LC neurons (Supplementary Fig. 3).

Consistent with the effect of L-lactate on electrical activity of LC neurons, L-lactate-induced NE release appeared independent of L-lactate caloric value, as pyruvate did not trigger NE release (Fig. 4d) and 4-CIN (100 μM) did not affect L-lactate-induced NE release (Fig. 4d).

### AC and PKA mediate L-lactate-evoked NE release

AC inhibition with SQ22536 (100 μM) completely blocked L-lactate-induced NE release (Fig. 4d). A PKA antagonist (H89; 10 μM) also prevented it, in contrast to a PLC blocker, U73122 (10 μM), which did not inhibit L-lactate-induced release of NE (Fig. 4d). Therefore, both the depolarizing effect of L-lactate and L-lactate-induced NE release critically depend on the activity of AC.

### L-lactate induces NE release in acute brain slices

To confirm that the ability of L-lactate to induce NE release is not a specific feature of the organotypic slices, which are prepared from young postnatal animals, we confirmed the key observations in acute slices prepared from either 30–33 day old or >2 months old (200–220 g) rats. At both ages L-lactate (400 μM) was a strong stimulant of NE release and the effects appeared greater than in cultured slices (Fig. 4e). Consistent with experiments in slice cultures, D-lactate and SQ22536 blocked the effect of L-lactate, and pyruvate evoked no NE release (Fig. 4e).

### Optogenetic activation of astrocytes triggers release of NE

We next tested whether selective activation of astrocytes results in L-lactate-mediated release of NE by LC neurons. In experiments presented below, we stimulated astrocytes using optoβ_2_AR^18^,^20^ (Fig. 4a) but stimulation with ChR2(H134R) yielded similar results (Supplementary Fig. 6).

**Figure 5:**
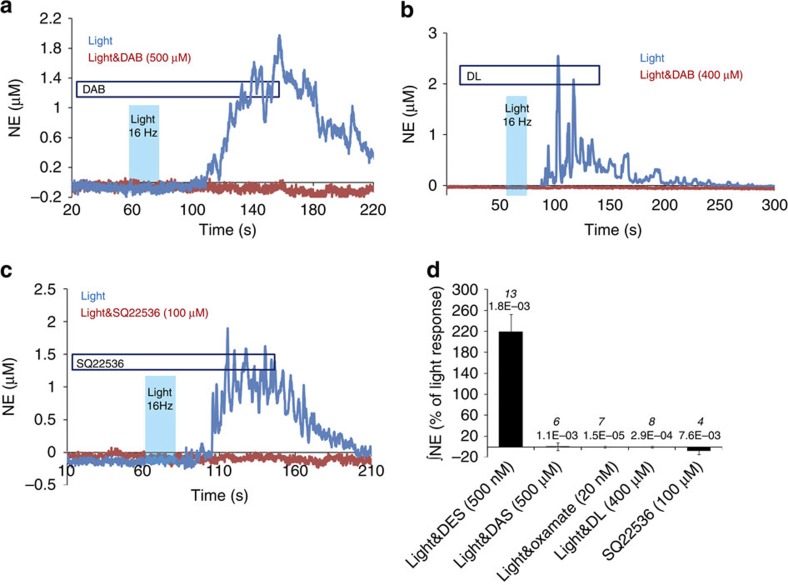
**Optogenetic activation of astrocytes using optoβ**_**2**_**AR triggers release of NE from LC neurons via L-lactate.** (**a**) A blocker of glycogen mobilization, DAB, abolishes NE release triggered by optogenetic activation of astrocytes. (**b**) D-lactate prevents NE release in response to optogenetic activation of astrocytes. (**c**) Inhibition of AC with SQ22536 suppresses NE release evoked by optogenetic activation of astrocytes. (**e**) Summary of pooled data from optogenetic stimulation of astrocytes. The NE signal was potentiated by DES (500 nM; Light: 113.9±15.5 μM s, *n*=25; Light&DES: 250.0±37.0 μM s, *n*=13). DAB (500 μM; Light: 91.0±18.7 μM s, *n*=7; Light&DAB: 0.8±6.7 μM s, *n*=6), oxamate (20 mM; Light: 56.6±8.0 μM s, *n*=11; Light&oxamate: −0.4±0.8 μM s, *n*=7), D-lactate (0.4 mM; Light: 76.1±16.6 μM s, *n*=13; Light&D-lactate: −0.6±1.1 μM s, *n*=8), and SQ22536 (100 μM; Light: 44.4±7.8 μM s, *n*=3; Light&SQ22536: −3.5±3.0 μM s, *n*=4) completely suppressed NE release evoked by optogenetic activation of astrocytes. Error bars depict s.e.m.; *P*-values are given above each column; Student’s unpaired *t*-test.

As mentioned previously, optogenetic activation of astrocytes triggers their acidification via L-lactate production (Supplementary Fig. 2b,c). We then proceeded with the experiments in organotypic-cultured slices where stimulation of optoβ_2_AR-expressing LC astrocytes with light (20 ms pulses, 16 Hz, 20 s train duration) resulted in a clear increase in NE oxidation currents, which were potentiated by DES (500 nM; Fig. 5; Supplementary Fig. 5b). Release of NE, optogenetically evoked via stimulation of astrocytic optoβ_2_AR, was abolished by DAB (500 μM, pre-incubated for >5 min), or by oxamate (20 mM; Fig. 5a,d). D-lactate (400 μM), as well as SQ22536 (100 μM), also completely blocked NE release (Fig. 5b–d).

Together these results suggest that activated astrocytes release L-lactate, which, in turn, triggers NE release from LC neurons via a cAMP-dependent signalling mechanism.

### Cardiovascular and EEG effects of L-lactate in LC *in vivo*

**Figure 6:**
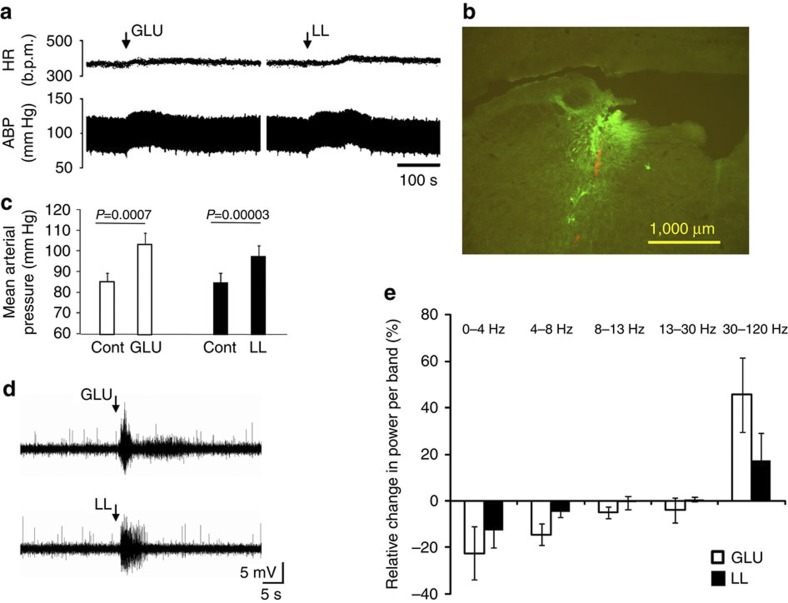
**Microinjections of L-lactate into the LC** ***in vivo*** **increase arterial blood pressure and EEG frequency.** (**a**) Representative trace from a urethane-anaesthetized rat where L-glutamate (GLU) and L-lactate were microinjected into the LC (recovery between applications at least 30 min). Top—in this particular case heart rate was increased by both stimulants, while in other locations we observed mild bradycardia. Lower—arterial blood pressure transiently increased in response to GLU and L-lactate microinjection. (**b**) Representative *post hoc* histological verification of the microinjection location. Following drug application, red beads were deposited in the tissue, which was then processed for dopamine-β-hydroxylase immuno-reactivity to reveal NEergic neurons (green). (**c**) Mean blood pressure changes in response to GLU and L-lactate (*n*=11 for each; Student’s paired *t*-test, error bars depict s.e.m.). (**d**) Example of an EEG response to GLU and L-lactate microinjections (recovery between applications approximately 30 min). We regularly observed a transient increase in total power and high frequency activity. (**e**) Relative changes in the power spectrum of EEG before and after microinjections of glutamate (*n*=4) and L-lactate (*n*=5) demonstrate a shift from lower to higher frequency bands. Error bars depict s.e.m.

On the basis of the previous observations, application of L-lactate to LC may be expected to evoke *in vivo* effects, which should be comparable to those of an established stimulant, for example GLU. This was tested in anesthetized and artificially ventilated rats. Microinjection of L-lactate (but not vehicle) into the LC evoked significant increases in arterial blood pressure (*+*13±2 mm Hg, *n*=11; *P*=2.9E–05, Student’s paired *t*-test; Fig. 6a–c). Moreover, L-lactate also evoked a strong increase in the power of high frequency bands of the power spectrum of electro-encephalogram (EEG; Fig. 6d,e). Qualitatively, the effects of L-lactate resembled those of GLU, injected at the same locations (increase in mean arterial blood pressure by 18±3 mm Hg, *n*=11; *P*=0.0007, Student’s paired *t*-test; Fig. 6a,c; EEG changes Fig. 6d,e). Both, L-lactate and GLU evoked variable heart rate changes, which were, however, always unidirectional at the same injection site (Fig. 6a). Injections of GLU outside LC were consistently ineffective as were injections of the buffer in LC or adjacent areas (Supplementary Fig. 7). These data confirm that L-lactate is excitatory to LC neurons *in vivo*.

## Discussion

**Figure 7:**
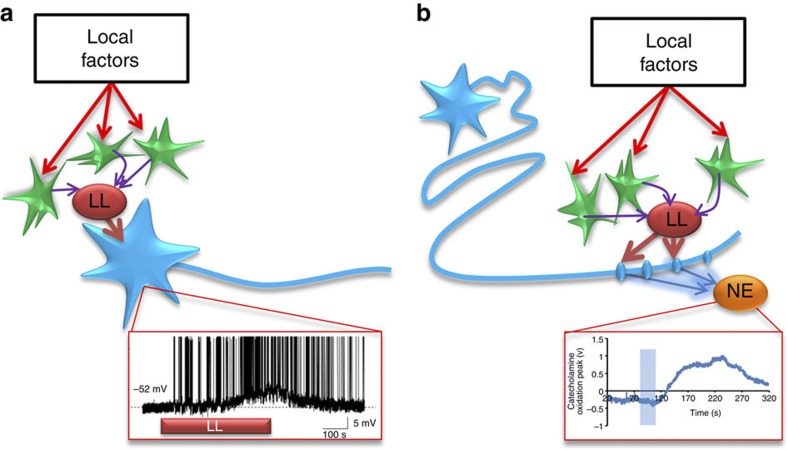
Putative mechanisms of L-lactate signalling between astrocytes and NEergic neurons. (**a**) Astrocytes (green) residing within the LC release L-lactate upon activation, which causes depolarization of the NEergic neurons (blue) and increases their firing rate (inset below). This could lead to both, increased NE release in the projection areas of LC neurons (for example cortex or hippocampus) and local somato-dendritic NE release within the LC which may primarily affect inhibitory auto-receptors. (**b**) Direct effects of L-lactate on the NE release machinery at the level of axonal varicosities may facilitate release of NE (inset below), irrespective of the frequency of incoming action potentials. In this way, NE release could be differentially regulated via a range of local signalling mechanisms in different projection areas innervated by the LC.

Here we demonstrate that exogenous L-lactate excites NEergic neurons in the LC electrically and triggers NE release. We also show that activated astrocytes release L-lactate, which excites adjacent NEergic neurons and stimulates release of NE. The effects of L-lactate are consistent with a role as a ‘glio-transmitter’ and cannot be explained by its use as an energy substrate for LC neurons. Since numerous factors can increase lactate production by astrocytes, this novel signalling pathway could potentially control NE transmission and affect a range of brain functions, which are regulated by inputs from LC (Fig. 7).

Depolarization of LC neurons, which follows optogenetic activation of local astrocytes, was blocked by oxamate or DAB, which prevents formation of L-lactate. D-lactate also blocked astrocyte-induced depolarization of LC neurons. Similarly, DAB, oxamate and D-lactate blocked NE release evoked by the activation of optoβ_2_AR-expressing astrocytes. Given that activation of astrocytes using either ChR2 or optoβ2AR led to the production of L-lactate (Supplementary Fig. 2) it is logic to conclude that this L-lactate was then released to excite NEergic neurons. Importantly, all the characteristics of the proposed L-lactate-mediated glia-neuronal signalling were consistent with the pharmacology of exogenous L-lactate.

L-lactate, pyruvate and beta-hydroxybutyrate can serve as complimentary fuels for central neurons^31^,^32^, but can L-lactate alter neuronal function when energy supply is ample? An earlier study reported excitatory effects of L-lactate on cultured neurons from the embryonic rat brain^33^, but in these experiments a supra-physiological concentration of L-lactate was used (20 mM) and solutions were not pH balanced, resulting in extra- and intracellular acidification (pH ~4.5). Two further studies reported acute effects of L-lactate on hypothalamic neurons, both supporting L-lactate’s role as a metabolic substrate. First, using patch electrodes containing no glucose and ATP, an excitatory effect of L-lactate on both glucose-inhibited and glucose-excited neurons mediated by via K_ATP_ channels was found^34^. Second, orexin-containing neurons decreased their firing rate after application of 4-CIN, indicating a requirement for L-lactate uptake^35^. In contrast, all tests conducted in this study consistently demonstrate that the excitatory effects of L-lactate on electrical activity of LC neurons and NE release are not related to use of L-lactate as a complimentary fuel. Conditions of all our experiments are such that neurons can be expected to have abundant sources of energy (high extracellular glucose, glucose and ATP in the pipette solution), and variations of extracellular glucose or level of oxygenation had no effect on the outcome of our tests (Fig. 3). Moreover, when 2 mM L-lactate was dialyzed into LC cells via a patch pipette, the membrane potential was unaffected but extracellular L-lactate was still excitatory (Fig. 2f). This condition should have collapsed or even reversed any inward gradient for L-lactate. Moreover, MCTs co-transport protons together with monocarboxylates and their ability to transport neutralized L-lactate at pH 7.4 must be limited. The high antagonistic efficacy of D-lactate in both patch clamp and NE release experiments is another testimony against the notion that L-lactate acts by entering LC neurons. D-lactate is a poor substrate to all known transporters as compared with L-lactate or pyruvate^36^,^37^,^38^. It follows that D-lactate may not have significantly affected entry of L-lactate into neurons when applied at concentrations 10 or even 100 times less than L-lactate, but nevertheless it powerfully suppressed L-lactate-induced effects. All other facts point in the same direction. Neither pyruvate nor acetate were effective in any of the tests, although both could be used as energy sources. A blocker of MCTs, 4-CIN, was completely ineffective, although in previous studies 4-CIN antagonized energy-mediated effects of L-lactate on orexin neurons^35^. Taken together, these data provide the first demonstration of a pH- and energy-independent excitatory effect of L-lactate on central neurons.

In both our patch clamp and FCV experiments, the threshold for L-lactate action was close to 0.2 mM. The EC50 of L-lactate was calculated to be 680 μM for patch clamp recordings (Hill slope ~0.7) and 456 μM for NE release. These values match the average physiological range of extracellular L-lactate found in the brain of a healthy rat (0.2–1 mM)^39^,^40^, but bursts of activity are likely to trigger steep local L-lactate gradients.

The effects of D-lactate documented here were unexpected and surprising. Indeed, in both patch clamp and FCV experiments, it acted as a potent inhibitor of L-lactate actions (Figs 1e,f, 2d, 3, 4c–e and 5b,d), this being consistent with the idea of D-lactate acting as a receptor antagonist rather than as a competitive substrate for a transporter. Since the excitatory effect of L-lactate does not require its uptake into LC neurons, it is logical to conclude that D-lactate interferes with L-lactate at a receptor site located on the extracellular side of the plasma membrane. Interestingly, in patch clamp experiments, D-lactate caused hyperpolarization suggestive of some basal tone within this signalling pathway under our conditions. In both patch clamp and FCV experiments, blocking AC with SQ22536 prevented the actions of L-lactate, implicating cAMP as a second messenger. Consistent with this idea, the PKA blocker H89 also prevented effects of L-lactate. In contrast, a PLC blocker U73122 was without effect. Because L-lactate-induced release of NE is little affected by TTX, it is possible that NE release evoked by L-lactate is, in part, independent of action potential firing of LC neurons. Previously, we demonstrated using carbon fibre micro-amperometry that TTX attenuates but not abolishes release of NE from identified varicosities of CA neurons in the rat brain^41^. Our earlier work in adrenal chromaffin cells showed that Ca^2+^ released from intracellular stores upon second messenger signalling can drive catecholamine release also in absence of depolarizations^42^. At the same time, NEergic neurons in slices typically have membrane potentials in the range of −50 to −65 mV (refs 21, 22). It is therefore equally possible that even mild depolarizations, which persist in the presence of TTX (as shown in Supplementary Fig. 3), are able to open voltage-gated Ca^2+^ channels, leading to release of NE, which can additionally be potentiated by cAMP-mediated signalling^43^.

Finally, *in vitro* results strongly suggested that application of L-lactate to the LC *in vivo* should trigger effects consistent with the activation of LC neurons and qualitatively (if not quantitatively) similar to those evoked by a standard excitatory neurotransmitter. Indeed, unilateral GLU and L-lactate into the same locations evoked unidirectional physiological responses, but only when the drugs were placed into LC. The choice of L-lactate dose for microinjection studies was based on the assumption that L-lactate should be used in concentrations/quantities equal or higher than those used for GLU, this being a conservative approach. Indeed, GLU receptors have nanomolar affinity, while our *in vitro* experiments required micromolar concentrations of L-lactate. At the same time, both molecules are subject to rapid uptake and removal from the site of injection. Both L-lactate and GLU evoked mild increases in blood pressure and triggered the expected changes in the EEG power spectrum with reductions in the low and marked increases in the high frequency bands. These results demonstrate that L-lactate has a profound excitatory effect on the LC.

Taken together, the characteristics of the L-lactate-mediated responses described above suggest the existence of a membrane receptor for L-lactate on LC neurons. However, it is highly unlikely that the hydrocarboxylic acid-1 receptor (HCA-1; previously known as GPR81, GPR104 or LACR1) described in adipose tissue could be responsible. HCA-1 is implicated in regulation of lipid breakdown^44^,^45^ and has an EC_50_ of ~5 mM (ref. 46), which is ~10 times higher than the estimates in the present experiments. Further, D-lactate is a weak agonist at HCA-1, while in our experiments it acted as antagonist or inverse agonist. The intracellular coupling mechanism of HCA-1 (GPR81) is incompatible with the effects described here as all published studies consistently demonstrated HCA-1 coupling to G_i_ proteins^44^,^45^,^46^,^47^. In neurons, G_i_-coupled receptors (for example GABA_B_ or α_2_-adrenergic receptors) inhibit adenylate cyclase, suppress neuronal activity and transmitter release, opposite to what was observed in this study. It is however possible that LC neurons might express either a variant of this receptor with a different coupling mechanism or yet another receptor that recognizes L-lactate and signals via activation of AC. Further studies are necessary to test this hypothesis.

What could be the role of L-lactate-mediated signalling to NEergic neurons? While numerous studies documented rapid L-lactate elevations evoked by various stimuli in the whole brain, very little is known about local L-lactate transients, which are highly likely to be prevalent because extracellular L-lactate is quickly taken up by both neurons and astrocytes and also drained from the CNS into the systemic circulation. It is therefore possible that local L-lactate concentrations are much more dynamic than is currently appreciated. It has also not been firmly established which mechanisms play the key role in control of glycogen breakdown by astrocytes *in vivo*, but NE receptors on astrocytes are ideally suited for this role^48^,^49^,^50^. It is therefore possible that L-lactate may act as a mediator of a positive feedback loop between NEergic varicosities and local astrocytes, whereby NE activates local astrocytes that further facilitate NE release via production of L-lactate. This mechanism could help to locally fine-tune NEergic input to numerous brain areas all innervated by the same source—the LC (Fig. 7). The physiological mechanisms that could recruit the L-lactate-mediated excitatory pathway between astrocytes and LC neurons are yet to be discovered.

## Methods

### AVV.PRSx8.RFP

All adenoviral vectors (AVV) were produced by standard homologous recombination^51^. To enable the visualization of NEergic neurons without concomitant opto-excitation, we expressed a monomeric red fluorescent protein^52^ under the control of the PRSx8 promoter, which is selectively active in NEergic neurons in the LC^53^.

### AVV.sPRSx8.ChIEF-tdTomato

To optically excite NEergic neurons, we expressed a fusion (kindly provided by Dr John Y. Lin, UCSD) between ChIEF, a light-sensitive cation channel with improved responses to high frequency stimulation, and a bright red fluorescent protein tdTomato, under the control of the PRSx8 promoter.

### AVV.sGFAP.ChR2(H134R)mKate

In patch clamp experiments, we used a fusion of a mutant of channelrhodopsin2, ChR2(H134R)^54^, with the red fluorescent protein Katushka1.3 (ref. 55). This allows excitation of astrocytes with blue light delivered by an additional laser or LED and visualization of transduced cells using green or yellow laser line of the confocal microscope. A compact transcriptionally amplified version of glial fibrillary acidic protein (GFAP) promoter was employed to enhance and target expression to astrocytes^56^,^57^. This AVV has previously been shown to elevate [Ca^2+^]_i_ levels and activate astrocytic signalling and transmitter release^4^. Although we did not further investigate the mechanism of L-lactate production triggered by this construct, it is important to state that it is highly unlikely to be a result of membrane depolarizations characteristic of ChR2-like constructs. Rather, it is most likely a consequence of an increase in [Ca^2+^]_i_ because the key regulator of glycogen breakdown, phosphorylase kinase can be directly activated by Ca^2+^-calmodulin, this pathway is well known and is described in various textbooks. In addition, several adenylyl cyclase isoforms that are expressed in the brain can be directly activated by a Ca^2+^-calmodulin^58^,^59^, for review see ref. 60. It is therefore feasible that Ca^2+^-calmodulin activates adenylyl-cyclase and cAMP thus formed acts by activating PKA, which phosphorylates phosphorylase kinase that in turn phosphorylates and activates glycogen phosphorylase.

### AVV.sGFAP.optoβ_2_AR

To activate astrocytic G-protein-coupled receptor-mediated intracellular signalling, we employed an opsin-β_2_-adrenergic receptor chimera (optoβ_2_AR), which is derived from extracellular and transmembrane parts of rhodopsin and the intracellular domains of the β_2_-adrenergic receptor^20^. Excitation of optoβ_2_AR by blue light recruits the G_s_PCR pathway, leading to the activation of adenylate cyclase (AC) and increase in cAMP (ref. 20). The astrocyte-selective promoter sGFAP (see above) was used to express optoβ_2_AR in astrocytes specifically.

The use of optoβ_2_AR only for FCV was determined by difficulties in handling cells expressing these constructs in patch clamp experiments. While establishing whole-cell configuration, it was essential to visualize the neurons for prolonged periods of time and with fairly intensive light. Compared with ChR2, we found it difficult to avoid unsolicited activation of astrocytes when optoβ_2_AR were used, possibly due to the wider excitation spectrum of these receptors. This construct also has a very narrow window for functional expression, while its overexpression is detrimental to astrocytes. Although these vectors are based on the same expression system as we used previously, for this study we have performed additional studies to verify lack of ‘leak’ expression in LC neurons with sGFAP-based vectors. Fourteen slices from four separate preparations were studied using confocal microscopy. Of 438 identified DBH-positive cells (LC neurons), only one was possibly double stained. Therefore, the vector system we use does not express optogenetic actuators in LC neurons (supplementary Fig. 8). Light did not evoke any consistent effects on neuronal membrane potential or NE release in slices not transduced with astrocyte-targeted AVV or expressing an inert construct (EGFP version CASE12), Supplementary Fig. 9.

### Organotypic-cultured brain slices

Experiments were performed in accordance with the UK Animals (Scientific Procedures) Act, 1986 and were approved by the local ethics committees.

Brainstem slice cultures were prepared as described previously^61^. In brief, Wistar rat pups of either sex (p7–9) were terminally anaesthetized with halothane and immediately decapitated. The brainstem was then removed and bathed in ice-cold dissection medium. Slices were cut at the level of the LC (200 μm thick) in cold (4 °C) sterile dissection solution using a vibrating microtome 7,000 (Campden Instruments, UK). Slices were then plated onto organotypic culture inserts (Millicell-CM, illipore) and supplied with 1 ml per /well plating medium containing the appropriate AVV, diluted to a titre of 10^8^ TU ml^–1^. Slices were kept at the interface between the feeding medium (pH 7.4) and humidified atmosphere (5% CO2, 37 °C). After 3 days, the plating medium was exchanged for supplemented Neurobasal medium (Gibco, UK), which was subsequently changed twice a week. Slice cultures were allowed to settle for 7–10 days before being used for experimentation to allow AVV-mediated expression to be fully established.

### Imaging of astrocytes

Primary cultures of Wistar rat pups (p2) cerebral astrocytes (adapted from^62^) transduced with AVV.sGFAP.optoβ_2_AR, seeded onto coverslips, were loaded with either Rhod-2AM (30 μM; Invitrogen, UK) or SNARF-5AM (30 μM; Invitrogen, UK) 1 h before visualization. Rhod-2AM was used as a Ca^2+^ indicator to avoid spectral interference with EGFP. The pH indicator SNARF-5 responds to acidification with a shift in its emission towards shorter wavelengths. Light was collected from two channels set to 550–580 nm (channel 1) and 620–700 nm (channel 2). Ratio of channel 1: channel 2 is indicative of a pH change.

Imaging experiments were carried out at 34 °C under continuous superfusion (150 ml h^–1^) with pH 7.4 HBS buffer in a tissue chamber mounted on a Leica-SP confocal microscope. Astrocytes were imaged in time-lapse mode (0.5 Hz) using the 543 nm laser line to excite Rhod-2 and SNARF-5. To activate optoβ_2_AR- or ChR2-transduced astrocytes, a 470 nm light-emitting diode-illumination system (Rapp Optoelectronic) coupled to the microscope via the epifluorescence port was employed. Changes of [Ca^2+^]_i_ in individual cells were assessed by changes in relative fluorescence intensity (*F*/*F*_0_).

### Ca^2+^ imaging and patch clamp of LC neurons

A cultured slice containing LC was transferred to a recording chamber mounted on an upright SP-2 confocal microscope (Leica, Germany) and continuously superfused with HEPES-buffered solution (HBS; in mM: NaCl 137, KCl 5.4, Na_2_HPO_4_ 0.25, KH_2_PO_4_ 0.44, CaCl_2_ 1.3, MgSO_4_ 1.0, NaHCO_3_ 4.2, HEPES 10, Glucose 5.5; pH 7.4; 34±1 °C). Current-clamp whole-cell recordings were performed at a 10 kHz sampling rate using an AxoClamp 2B amplifier (Molecular Devices), a 1401 interface and Spike 2 software (both from CED, Cambridge, UK). Recording pipettes were pulled with a vertical puller (Narishige PC-10) to 3–5 mΩ resistance. The pipette solution consisted of (in mM): potassium gluconate 130, HEPES 10, EGTA 11, NaCl 4, MgCl_2_ 2, CaCl_2_ 1, ATP 2, GTP 1 and glucose 5. For intracellular L-lactate application, 2 mM L-lactate (pH 7.4) was introduced to the cell by a recording pipette with slightly higher impedance (8–9 mΩ) to slow down L-lactate diffusion and aid visualization of possible time dependent effects of intracellular L-lactate. In these experiments a red-coloured dye was added to the pipette (usually SNARF-5) to confirm successful dialysis of the patched neuron. For Ca^2+^ imaging, cells were loaded with the Ca^2+^ indicator Rhod-2 by recording pipette. The intracellular solution used in these experiments contained the following (in mM): potassium gluconate 130, HEPES 10, EGTA 5.5, NaCl 4, MgCl_2_ 2, CaCl_2_ 1, ATP 2, GTP 0.5, glucose 5 and Rhod-2 0.5. Changes in [Ca^2+^]_i_ were assessed by relative fluorescence intensity (F/F_0_). Rhod-2 was excited using 568 nm laser line, and Rhod-2-emitted fluorescence was sampled at 580–640 nm. For further details see ref. 26. [Ca^2+^]i responses in LC astrocytes were visualized using a genetically encoded Ca^2+^ indicator Case12 expressed via an AVV with enhanced GFAP promoter. To boost the expression of the transgenes, the construct employs coordinated co-expression of a chimeric transcriptional activator NFκB-GAL4 (ref. 4).

### FCV of extracellular NE

Changes in NE tissue concentrations were measured by FCV using a Millar voltammeter (PD Systems International, UK) and carbon fiber electrodes (CFE; CF10-100 obtained from World Precision Instruments, USA, or produced in house with comparable sensitivity). A symmetric, triangular wave with a range of −0.7 V to 1.3 V (5 ms; 8 Hz) was applied through the CFE to oxidize (at around +0.65 V to +1.0V) and reduce (−0.4 V to −0.6 V) NE (Fig. 5a). Slice cultures containing the LC and transduced with selected AVVs (AVV.sGFAP.optoβ_2_AR, AVV.PRSx8.RFP, AVV.sPRSx8.ChIEF-tdTomato) were immobilized in the recording chamber by a mesh and continuously superfused with HBS (pH 7.4, 34 °C).

The CFE was initially placed in a non-transduced area of the slice and allowed to cycle for 1 h to stabilize the baseline before recording, after which it was transferred to the LC region. Optogenetic stimulation was applied to the LC through an optical fiber (0.7 mm Ø) positioned 10–12 mm above the slice and connected to a PhoxX 445 nm laser (Omicron, Germany).

Before recording each epoch, the background electrochemical signal was subtracted using the ‘refresh’ function of the voltammeter. The resulting Faradaic signal data were acquired and analysed through a Micro1401II interface using Spike2 software (both from CED, Cambridge, UK) with scripts written in house. The NE oxidation peak (between +0.65 V and +1.0 V on the ascending scan) was plotted against time and the integrated NE oxidation in response to L-lactate or light stimulation was determined as V × s. CFEs were retracted from the tissue at the end of experiments and calibrated against NE (0.5 μM) to allow calculation of total release as μM × s. Independently of the electrode sensitivity, we can expect variability in the number of local release sites around any recording position, thus resulting in variation of measured NE volume transmission between recording sites or slices. Therefore data sets including the relevant and comparable control responses were pooled for evaluation of effects.

### *In vivo* experiments

Eleven male Sprague–Dawley rats (300–340 *g*, 2.5–3 months of age) were anesthetized with urethane (1.6 g kg^−1^, I.P.). Adequate anaesthesia was ensured by maintaining stable levels of arterial blood pressure and heart rate. The femoral artery and vein were cannulated for measurement of arterial blood pressure and administration of anesthetic, respectively. The trachea was cannulated, and the animal was ventilated with a mixture of 50% oxygen and 50% nitrogen using a positive pressure ventilator (Harvard rodent ventilator, model 683) with a tidal volume of ~2 ml and a ventilator frequency similar to spontaneous frequency (~60 strokes min^−1^). The body temperature was maintained with a servo-controlled heating pad at 37.0±0.2 °C.

The animal was placed in a stereotaxic frame, and a small hole was drilled in the occipital bone overlying the left LC (AP −9.8 mm; ML −1.2 mm from bregma). For EEG recordings, two small holes were drilled on the left side of the skull above the somatosensory cortex. Stainless steel electrodes were placed in the contact with the cortical surface and secured in place with dental cement.

A three-barrelled glass micropipette (tip size 20–25 μm) was placed in the LC (9.7 mm caudal, 1.15 mm lateral from bregma and 8–8.5 mm ventral from the surface of the skull). The barrels of the micropipette contained L-glutamate (500 mM, pH 7.4), L-lactate (500 mM, pH 7.4) and saline containing 5% of fluorescent beads (Invitrogen). Drugs microinjected from pipettes in small volumes are subject to very rapid dilution and therefore activate a much larger area than the actual volume of injection. In addition, metabolites such as glutamate of L-lactate are taken up by the cells and also eliminated from the tissue into the bloodstream. On the basis of the effective concentrations of L-lactate *in vitro* established in this work, L-lactate could be applied at concentrations much higher than those of L-glutamate because L-glutamate receptors have high affinity, while the putative L-lactate receptor must have low affinity. Nevertheless we decided not to increase it further to avoid possible complications due to an osmotic effect. Microinjections were made using pressure over a period of 5–10 s and were monitored by observing the fluid level using a dissecting microscope with a calibrated micrometer disk. First, a unilateral microinjection of L-glutamate (40 nl) was made to document the effect of LC activation on cardiovascular variables and EEG. After a recovery period of 30–40 min, L-lactate was microinjected (40 nl) into the same LC site. The sites of microinjections were marked by injection of fluorescent beads, identified *Post hoc* histologically and mapped using a stereotaxic atlas^63^. The EEG signal was amplified via a differential amplifier (Digitimer, USA) without additional filtering.

Recordings were processed using a Power1401 interface and analyzed with *Spike2* software (CED). Measurements of mean arterial blood pressure and heart rate were taken before and at the peak of the evoked responses and were compared using Student’s paired two-tailed *t*-test.

The power spectrum of the EEG signal was analyzed using Spike2 software using a custom-written script. 50 (±2) Hz band was excluded to avoid skewing by potential AC interference. L-lactate- and L-glutamate-induced changes in <4 Hz (delta), 4–8 Hz (theta), 8–13 Hz (alpha), 8–30 Hz (beta) and >30 (gamma) power bands were determined and compared within individual experiments.

### *Post hoc* histology

Fixed brains were sectioned and immunostained for dopamine-β-hydroxylase, an established marker for NE neurons. Monoclonal primary antibody was used (MAB308, Millipore, 1:1,000) followed by a goat anti mouse-Alexa 488 (Invitrogen, 1:500).

### Drugs

The following drugs were obtained from Tocris: MRS 2179, CNQX, APV, indomethacin, tetrodotoxin, SQ22536, U73122. All other chemicals were from Sigma.

### Statistics

Pooled data were expressed as average ±s.e.m. Groups were compared using Student’s *t*-test, paired or unpaired as indicated. Microsoft Excel was used for data processing. GraphPad Prizm was used to calculate EC50 values for concentration-response curves.

## Author contributions

F.T. and S.L. performed a part of the experiments in slices, analyzed results, prepared some of the figures. A.K. performed some of the *in vivo* experiments. J.F.R.P. edited and commented on the manuscript. A.V.G. designed and performed *in vivo* experiments, evaluated results, wrote parts of the manuscript. S.K. and A.G.T. designed the study, evaluated some of the results, wrote parts of the manuscript.

## Additional information

**How to cite this article:** Tang, F. *et al*. Lactate-mediated glia-neuronal signalling in the mammalian brain. *Nat. Commun.* 5:3284 doi: 10.1038/ncomms4284 (2014).

## Supplementary Material

Supplementary Information Supplementary Figures 1-9
