# Supplementary material for: Lactate-mediated glia-neuronal signalling in the mammalian brain
Source: Nat Commun. 2014 Feb 11;5:3284. doi: 10.1038/ncomms4284 (PMC3926012; doi:10.1038/ncomms4284)
Supplement: Supplementary Information — Supplementary Figures 1-9 [file ncomms4284-s1.pdf]

Supplementary Figure 1

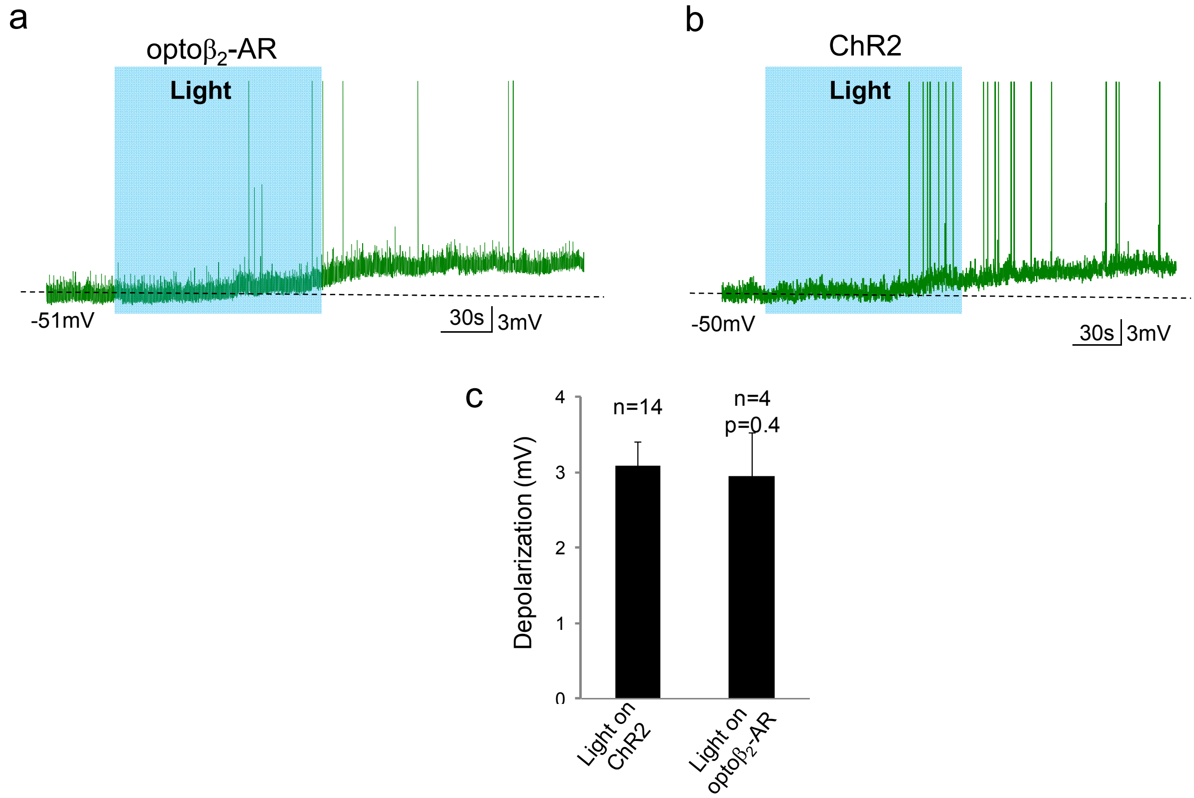

Both optogenetic actuators, optoβ_2AR and ChR2, were effective in stimulating astrocytes and evoking depolarizations in LC neurons.

It is acknowledged that stimulation via ChR2 activation does not mimic any specific physiological effect characteristic to astrocytes but it does lead to release of ATP and increases in $[Ca^{2+}]i$ in these cells, the two events which are firmly associated with "excitation" of astrocytes. The use of mainly ChR2 in the first part of the study was ultimately determined by the high light sensitivity of optoβ_2AR which posed difficulties for patch clamp experiments.

a – Light-activated astrocytes expressing optoβ_2AR depolarized an LC neuron; representative trace.

b - Astrocytes expressing ChR2 were light-activated and evoked depolarization and firing of action potentials in an LC neuron, representative trace; see also Fig 1b

c – Pooled data comparing ChR2 and optoβ_2AR-mediated depolarizations in LC neurons. Action potentials truncated. n refers to numbers of individual slices (cells). Paired Students t-test versus pre-stimulus membrane potential. Error bars depict S.E.M.

Supplementary Figure 2

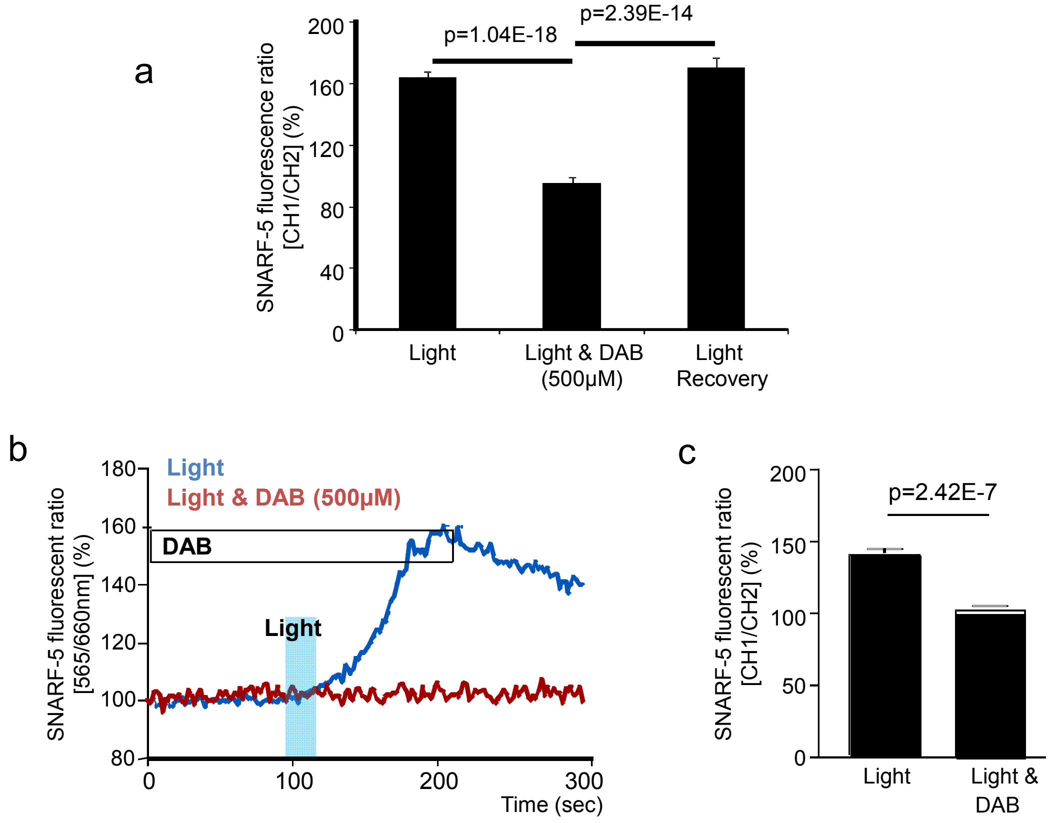

Optogenetic activation of astrocytes results in acidification which is due to intracellular LL accumulation.

a – Light stimulation of ChR2(H134R)-expressing cultured astrocytes caused a shift in SNARF-5 emission ratio towards short wavelengths (CH1/CH2 ratio: 161.7±5.1%, n=58 cells) , indicative of acidification. When cultures were pre-incubated in DAB (500µM), this acidification was abolished (95.3±4.2%, n=29) but the effect could be reproduced following wash out of DAB (166.7±5.4%, n=29). Student's unpaired t test.

b – Example of an experiment where light stimulation of optoβ_2AR-expressing cultured astrocytes caused a similar shift in SNARF-5 emission ratio, consistent with acidification, which was prevented by pre-incubation with DAB (500µM). Individual traces were normalized to their own control ratio.

c – Summary of data for activation of astrocytes using optoβ_2AR (CH1/CH2 ratio: 144±2.2%, n=58). Acidification was prevented by DAB (101.3±0.7%, n=32). Student's unpaired t-test. Error bars depict S.E.M.

Supplementary Figure 3

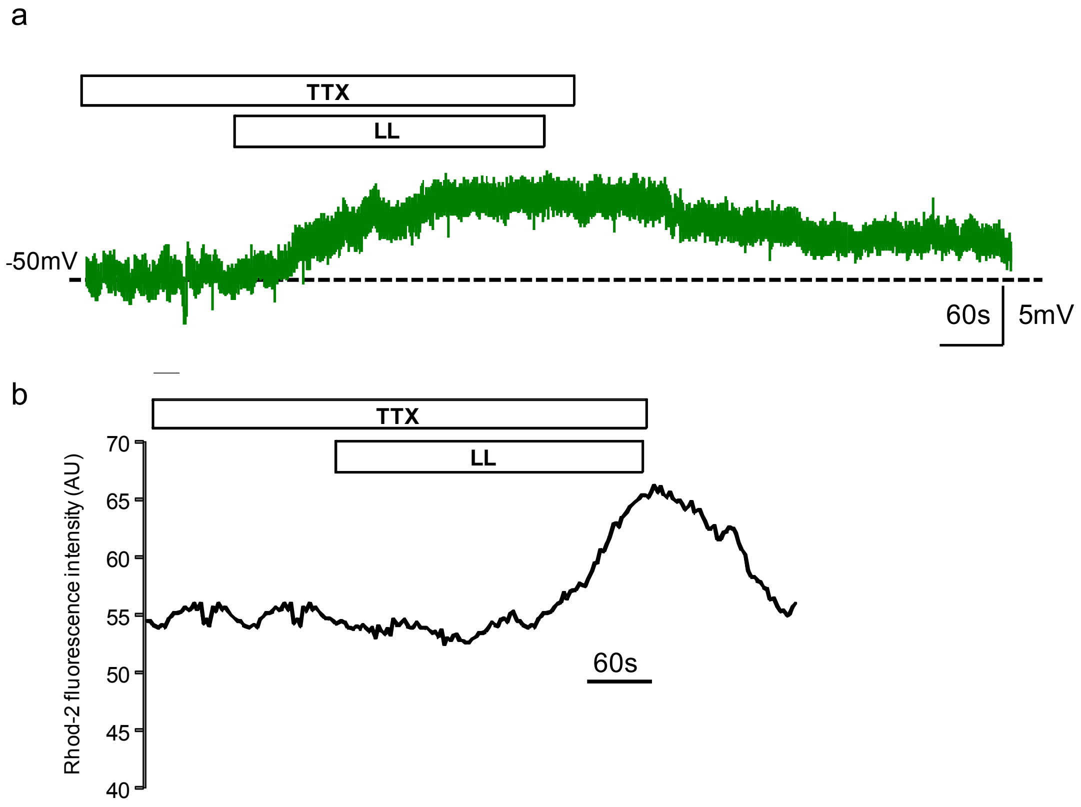

The excitatory effects of LL on LC neurons are largely preserved in TTX (separate examples).

a – TTX (1 μM) does not prevent LL (2mM) induced depolarization in an LC neuron

b – TTX does not prevent LL-induced [Ca^{2+}]i increase in an LC neuron

Supplementary Figure 4

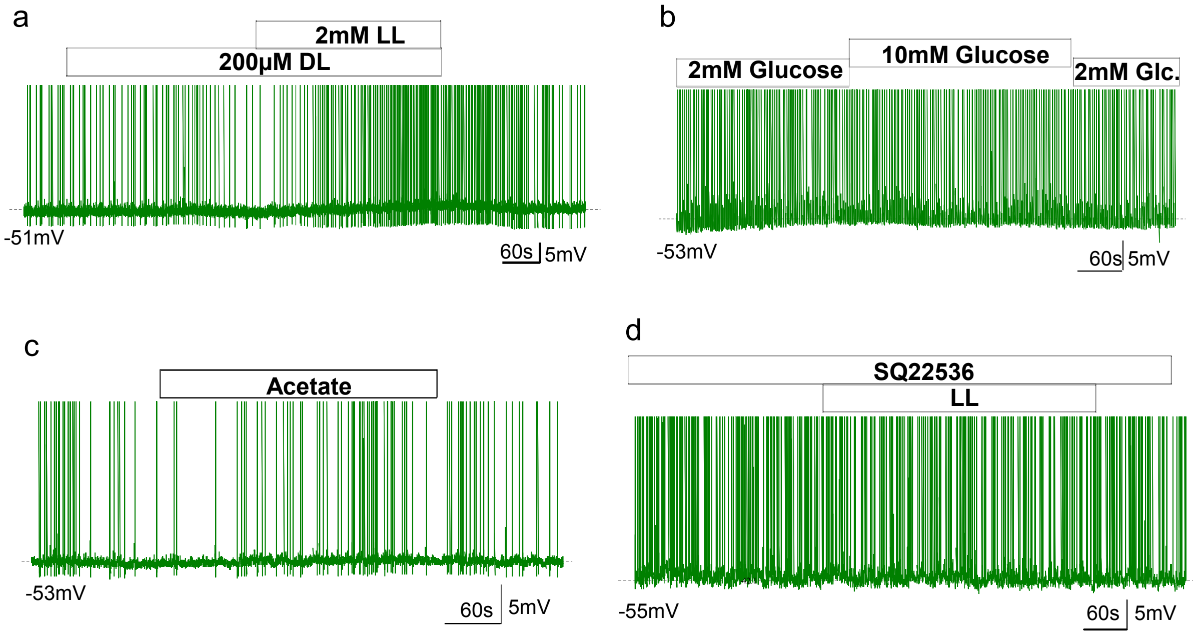

Representative traces for the data summarized in Fig 3

a – A representative trace showing that the depolarizing effect of LL on LC neurons is strongly attenuated by a 10 times lower concentration of DL, although DL is less well transported by MCTs than LL. Note that the baseline firing rate of this cell and other neurons shown here is 1Hz or lower as typical for many LC neurons.

b – An example to illustrate that change between 2 and 10 mM glucose has no measurable effect on the electrical activity of LC neurons under our experimental conditions. This argues against the need for extra ATP generation in these cells.

c - A trace from a typical experiment to illustrate that acetate (2mM, pH 7.4) has no effect on the firing rate and membrane potential of LC neurons

d - An example demonstrating that the depolarizing effect of LL is abolished in the presence of an inhibitor of adenylyl cyclase SQ 22536 (100 μM). This suggests involvement of a Gs-coupled signaling mechanism.

Supplementary Figure 5

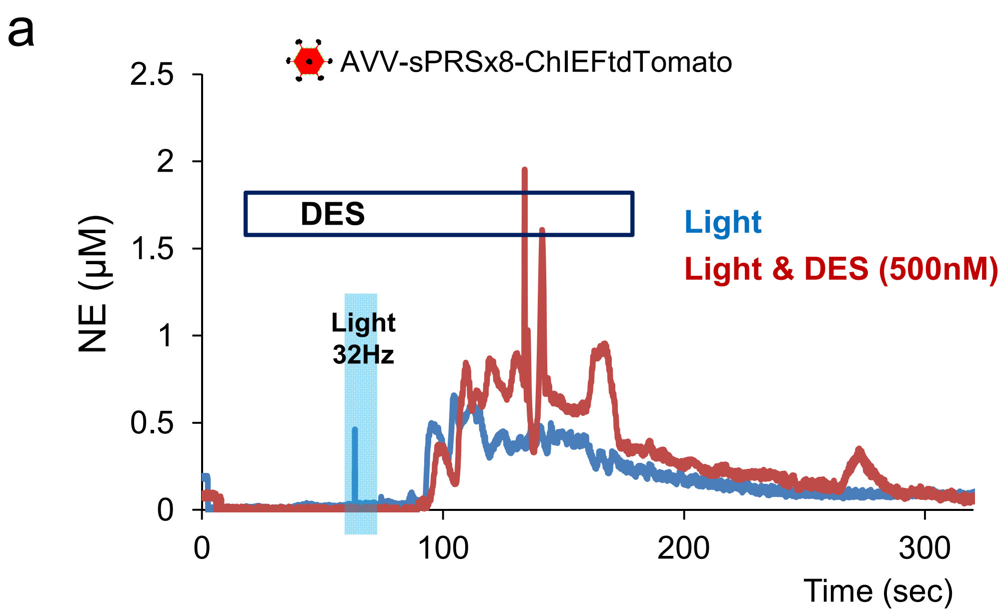

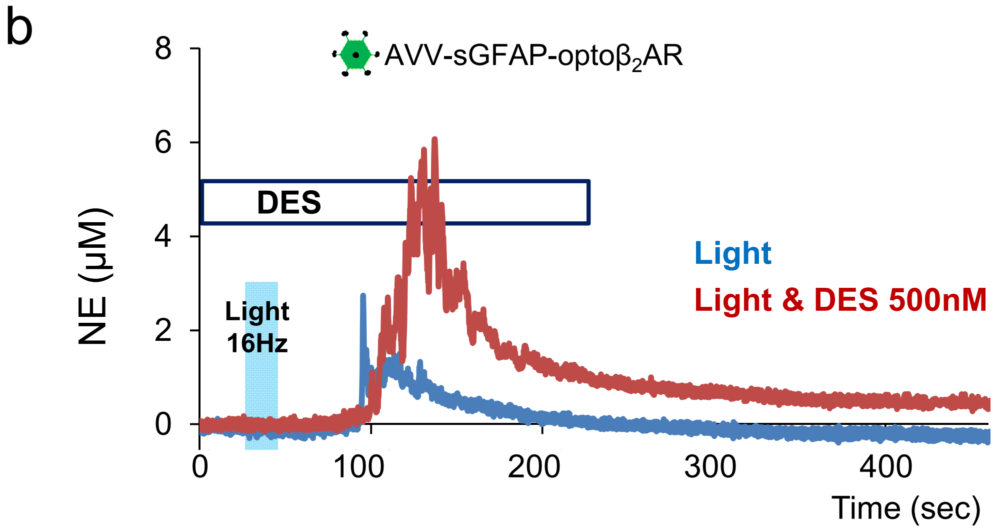

The NE reuptake blocker desipramine (DES, 500 nM) increases and prolongs voltammetric signals, confirming the identity of the oxidized substance as NE.

a – Direct optogenetic activation of NEergic neurons via expression of the actuator ChIEF evoked NE release in an organotypic slice containing the LC. The NE signal was potentiated by 500 nM DES (M ±S.E.M.; Light: 38.7±9.6 µMxsec, n=30; Light&DES: 62.5±13.5 µMxsec, n=7; p=0.09).

b – Indirect optogenetic activation of NEergic neurons via expression of optoβ_2AR in astrocytes elicits NE release. Again, the NE signal is potentiated in the presence of DES (500 nM; Light: 113.9±15.5 µMxsec, n=25; Light&DES: 250.0±37.0 µMxsec, n=13, p=0.002).

Supplementary Figure 6

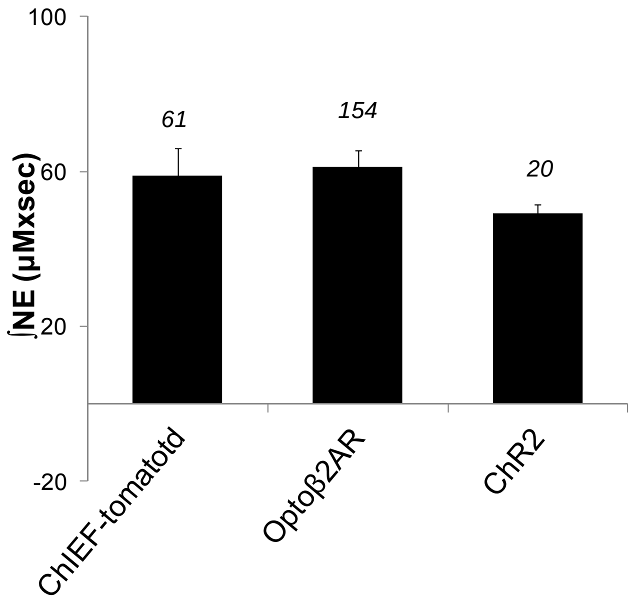

Optogenetic stimulation of LC neurons directly via ChIEF illumination, or indirectly via light-activation of astrocytes expressing either optoβ_2AR or ChR2 evokes NE release.

M ± S.E.M. Values above the bars refer to the number of individual experiments with each actuator.

Supplementary Figure 7

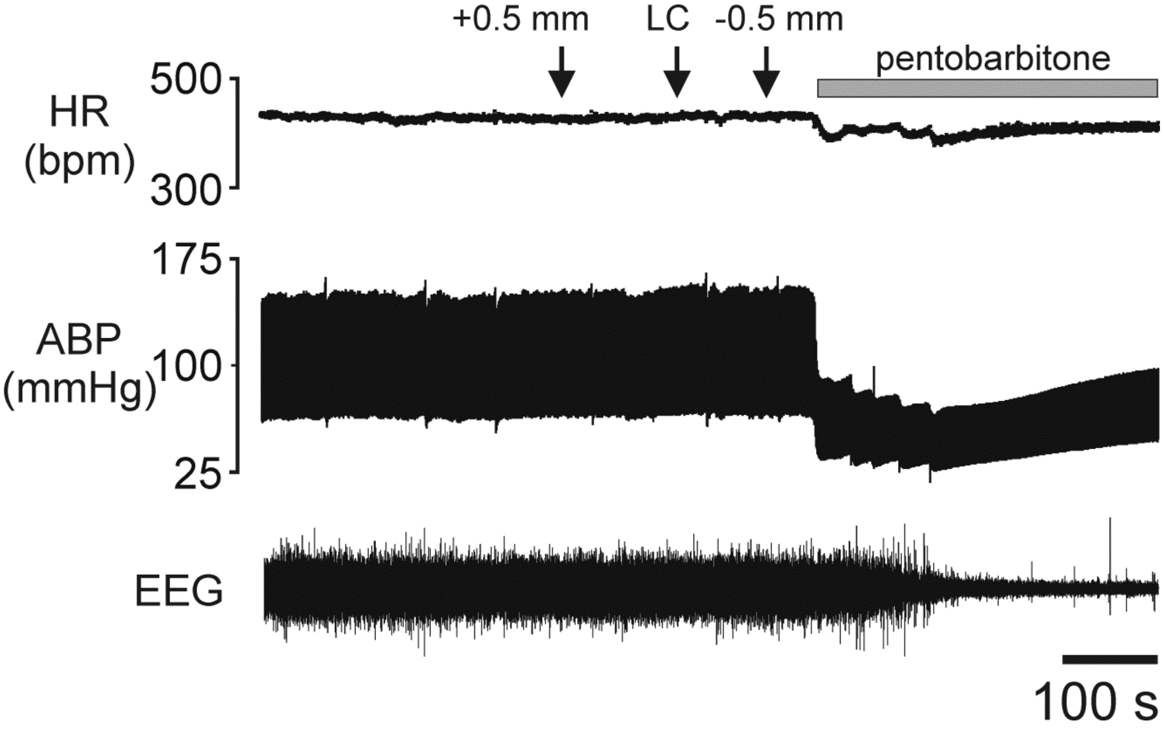

Microinjections of buffer solution into LC or adjacent areas was without any noticeable effects (control trace).

Injection of buffer solution alone had no effect in either LC or areas above and below it. Pentobarbitone i.v. was used as a positive control at the end of the experiments and caused a dramatic drop in the amplitude of the EEG, arterial blood pressure (ABP) and heart rate (HR).

Supplementary Figure 8

Viral vectors used for delivery of astrocyte-targeted optogenetic actuators do not express genes in catecholaminergic (dopamine β hydroxylase positive; DBH) LC neurons

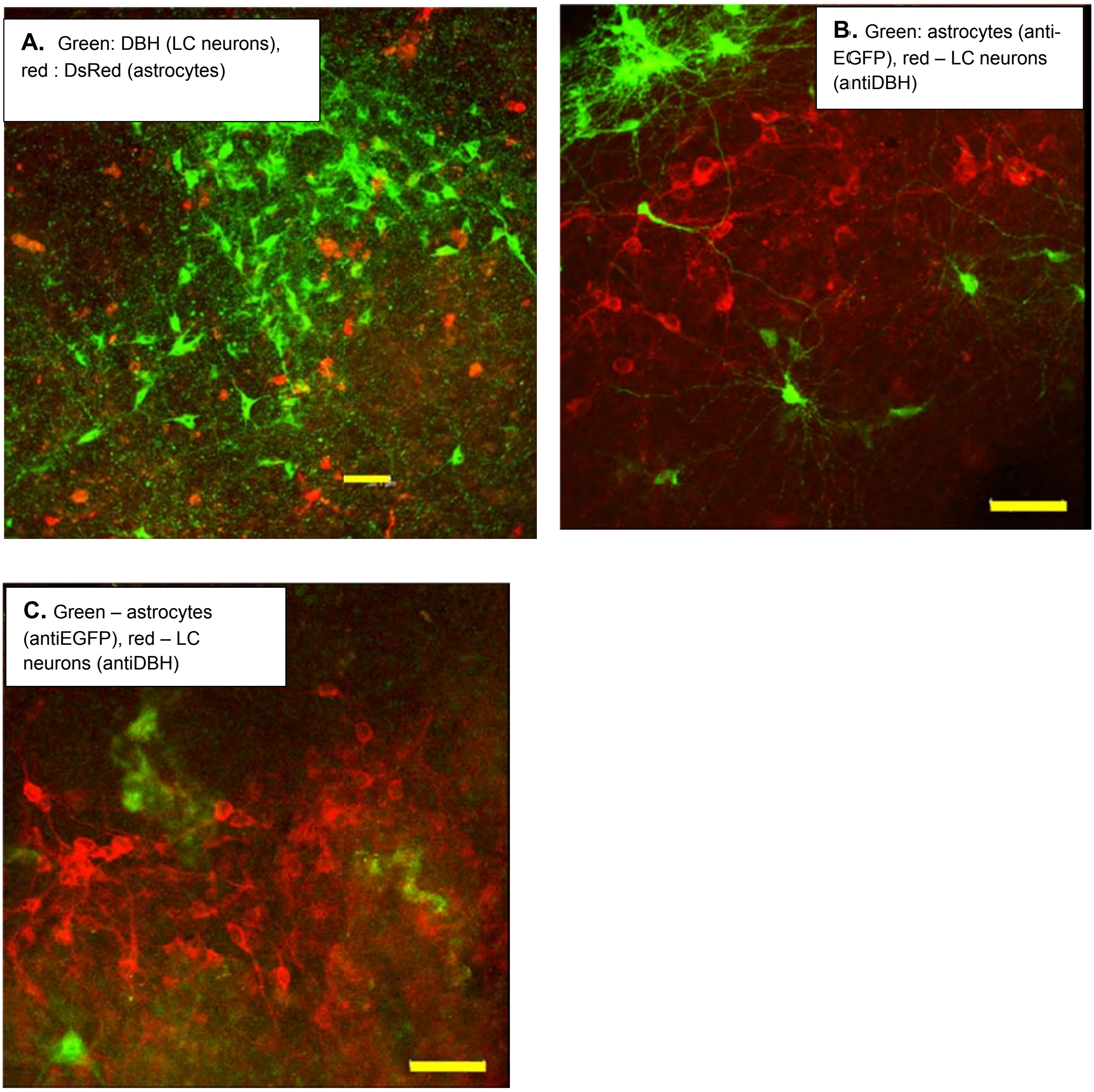

Four AVV with the same sGFAP promoter system were tested (AVV.sGFAP.ChR2(H134R)mKate, AVV.sGFAP.optoβ_2AR, AVV.sGFAP.DsRed2 and AVV.sGFAP.Case12). Of 438 identified DBH-positive cells (LC neurons) only one was possibly double-stained. Therefore the vector system we use does not express optogenetic actuators in LC neurons.

A. In this example astrocytes in organotypic slices containing LC were targeted with AAV.sGFAP.DsRed2 to express a bright red fluorescent protein. Slices were then

fixed and immunostained for DBH (Millipore MAB308) using a secondary fluorescent antibody coupled to a green fluorophore Alexa 488 (Invitrogen). Note that the density of red fluorescent astrocytes is much less than can be expected within this volume of tissue, this reflects the fact that our experimental conditions never lead to transduction of all potentially targetable astrocytes. Also note that only the transduced astrocytes are targets for light stimulation when stimulated with blue light. Therefore there are only a few astrocytes next to each individual LC neuron which can be realistically expected to be activated by light.

Scale bars on all panels: 50 μm.

B. Astrocytes in organotypic slices containing LC were targeted with an AVV with sGFAP promoter to express an EGFP variant Case12 and double immunostained for EGFP (Invitrogen) coupled to green secondary antibody (Invitrogen, Alexa-488). LC neurons were stained with anti-DBH antibody coupled to a red secondary antibody (Invitrogen, Alexa 594). As we often observe, most of the gene expressing astrocytes concentrate to the periphery of the cluster of the DBH-positive cells (LC neurons) while within the nucleus itself their density is typically much lower. This could explain latency in some of the responses which would be expected due to diffusion of NE. This image also illustrates that typically we are only activating a few astrocytes next to each individual neuron. Finally, there is no color overlap, indicating lack of “leak” expression of Case12 (EGFP) in LC neurons even though the EGFP signal has been amplified by antibodies.

C. Astrocytes in organotypic slices containing LC were targeted with AVV.sGFAP.optoβ_2AR and immunostained for EGFP (both antibodies from Invitrogen, green secondary antibody is Alexa 488 conjugated) and DBH (same as in **b**). Native EGFP fluorescence may not be visualized with this construct, probably because at high level of expression it becomes cytotoxic. Diffuse green fluorescence is present in several cells located largely at the periphery of the cluster of LC neurons. There is no noticeable color overlap indicating lack of “leak” expression in LC neurons even though the EGFP signal has been amplified by antibodies.

Supplementary Figure 9

Examples to illustrate that blue light does not electrically activate LC neurons and does not evoke NE release as registered using FCV in slices which have not been transduced to express optogenetic actuators.

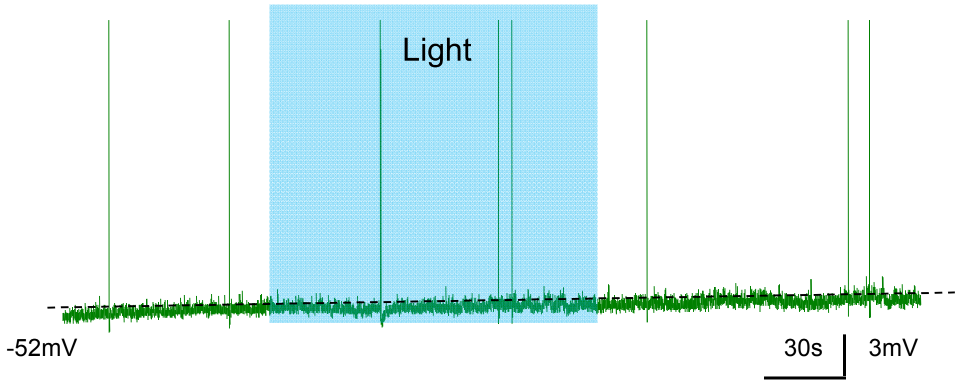

a - Astrocytes were transduced to express an EGFP variant Case12 and transduced with AVV.sPRSx8.DsRed to visualize LC neurons. LC neurons were patched exactly as in other experiments described in this study and slices were stimulated with blue light as described in the paper. Light produced no consistent changes in activity of LC neurons in these slices (n>7).

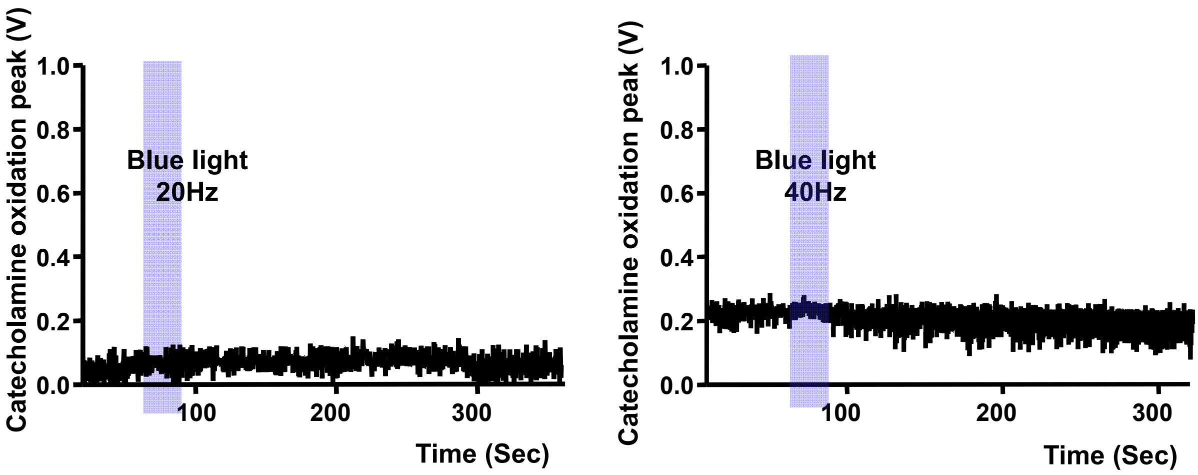

b - Traces to illustrate that in organotypic slices which have not been transduced with optogenetic actuators blue light (20 or 40 Hz, different experiments) does not evoke release of NE. Raw, unfiltered currents plotted against time are shown.
